# The bestersell effect: Nuances in positional encoding of morphemes in visual word recognition

**DOI:** 10.3758/s13423-025-02693-7

**Published:** 2025-04-29

**Authors:** Jasmine Spencer, Hasibe Kahraman, Elisabeth Beyersmann

**Affiliations:** 1 School of Psychological Sciences, Macquarie University, Australian Hearing Hub, 16 University Avenue, Sydney, NSW 2109 Australia; 2 Macquarie University Centre for Reading, Macquarie University, Sydney, Australia

## Abstract

Previous studies have confirmed stem morphemes (e.g., *book*) are identified in any position (e.g., in both *bookmark* and *textbook*) but prefixes and suffixes (e.g., *re*- in *replay* and -*er* in *player*) cannot be recognized when moved from their typical word-initial or word-final locations. However, English words with multiple affixes (e.g., *unresolved, mindfulness*) suggest there must be further nuance to the positional constraints imposed on affixes in the reading system to facilitate cases where affixes occur in atypical locations but still convey meaning. We used two lexical decision experiments (*N* = 90 native English-speaking participants each) to investigate the positional encoding of mid-embedded suffixes. In Experiment 1, transposed tri-morphemic nonwords ending in a chain of two suffixes (e.g., *spitenessful* [derived from *spitefulness*]), and transposed nonwords with string-initial suffixes (e.g., *fulyouthness* [derived from *youthfulness*]) were compared against orthographic controls (e.g., *spitementdom*/*domyouthment*). In Experiment 2, transposed tri-morphemic nonwords ending in a stem (e.g., *bestersell* [derived from *bestseller*]) and transposed nonwords with string-initial suffixes (e.g., *erwalksleep* [derived from *sleepwalker*]) were compared against orthographic controls (e.g., *bestalsell*/*enwalksleep*). Across both experiments, the results revealed a significantly larger morpheme transposition effect relative to controls for the mid-embedded compared with the string-initial suffix conditions. Items like *bestersell* activated the corresponding lexical representation of “bestseller” and made it more difficult to reject the target nonword, revealing that suffixes are not as strictly positionally encoded as previously assumed. These findings challenge existing predictions of positional requirements for affixes and provide evidence calling for more nuanced theoretical models of morphological processing.

Processing morphemes, the smallest meaningful units within words, is fundamental to our comprehension of language. Most English words exhibit morphological complexity, with constituent morphemes influencing visual word recognition (e.g., Grainger et al., 1991, 2021; Longtin & Meunier, 2005; Rastle et al., 2004; Solaja & Crepaldi, 2024). While monomorphemic word processing has been more extensively studied (e.g., Grainger, 2008, 2022; Norris, 2013; Rastle, 2007), including through computational models of reading (for a review, see Snowling et al., 2022), the processing of morphologically complex words remains an area where reading theories are underspecified and thus warrants further investigation (for a recent review, see Stevens & Plaut, 2022). 

One outstanding question is how the reading system handles the positional constraints of morpheme processing. This question is particularly important in the context of Indo-European languages, such as English, which follow a fixed, linear prefix-stem-suffix morpheme structure (e.g., *un*-*load*-*ing*). Prefix-stem-suffix morphologies have prefixes always occurring before the stem (e.g., *un*-*load*), and suffixes following the stem (e.g., *load*-*ing*). The linear nature of morpheme position in English is best illustrated when directly compared against nonlinear morphological structures. Nonlinear morphological structures, like the Semitic root-and-pattern morphology of Hebrew and Arabic or the polysynthetic structure in Inuktitut, involve intercalating morphemes in complex ways that do not follow the rigid prefix-root-suffix order found in English. Based on (i) these fundamental linguistic constraints in morpheme position and (ii) evidence pointing to the important role of statistical regularities in visual word recognition (Carr et al., 2024; Lelonkiewicz et al., 2023), it is likely that readers of linear morphologies are sensitive to the predicted morpheme positions in their reading, which is what the current study aimed to investigate. 

Indeed, evidence from lexical decision tasks has revealed evidence for the important role of morpheme position during visual word recognition. Readers can easily recognize *play* in *playful* and *multiplayer* and *replay*, indicating the position-independent or ‘free’ nature of stems (Beyersmann et al., 2016, 2018; Heathcote et al., 2018). For instance, results from masked primed lexical decision have revealed significant priming effects for compound word targets that have a constituent in common with a related compound prime regardless of the position of this shared stem morpheme (e.g., both *postman* and *milkshake* facilitate the lexical decision of the target word *milkman*; Duñabeitia et al., 2009). Further evidence for the position-independent recognition of stem morphemes comes from studies specifically probing positional constraints using the morpheme transposition paradigm. For example, Crepaldi et al. (2013) found that morpheme-transpositions derived from real compound words (e.g., *washcar* from *carwash*) had longer rejection times in unprimed lexical decision tasks than control items (e.g., *luckcar*), indicating that *washcar* was able to activate the representation of *carwash* despite the stem constituents appearing in transposed positions within the letter string. The same authors also used a masked primed lexical decision task, showing that morphemically transposed items primed the original words from which they were derived (e.g., *firecamp*–*CAMPFIRE*) relative to unrelated controls. Crucially, this same priming did not occur for monomorphemic conditions (e.g., *rickmave*–*MAVERICK*), suggesting that the morpheme transposition effect was not due to orthographic prime–target overlap (Crepaldi et al., 2013). 

In contrast to transposed compound words, the same pattern of interference effects has not been found for transposed suffixed items (e.g., *istunion* vs. *irtunion*). Moreover, primes that shared a suffix with the target but at their onset also failed to produce a morpheme transposition effect (e.g., *nessbolt*–*kindness*), suggesting that suffixes were not recognized outside their typical position at the end of a letter string (Crepaldi et al., 2010, 2016; Duñabeitia et al., 2007). This finding was further confirmed and extended to prefixes by a recent morpheme-transposition study that directly compared stems and affixes within the same experiment (Spencer et al., 2024). Spencer and colleagues (2024) showed that transposed compound items produced longer reaction times (RTs) and higher error rates (ERs) compared with their orthographic controls that do not map onto a real word (e.g., *dreamday* vs. *shadeday*), while there was no increased difficulty for transposed suffixed items (e.g., *fulpain* vs. *dompain*) or transposed prefixed items (e.g., *qualifydis* vs. *qualifymis*) relative to controls (Spencer et al., 2024). Spencer et al.’s findings suggest that, compared with the recognition of stem morphemes, readers do not employ the same kind of broad search for affix detection. Such a mechanism prevents the erroneous recognition of prefixes in word final position (e.g., *re*- in *there*) or suffixes in word initial position (e.g., -*er* in *error*). 

This nuance in positional constraints has begun to be incorporated in recent theories of morphological processing. Theoretical models of morphological processing broadly fall into two camps. Decompositional models predict that words are segmented into representations of their constituent morphemes (for a review, see Marelli et al., 2020), whereas distributional models predict that “morphological sensitivity arises from the tuning of finer-grained representations to useful statistical regularities in the form-to-meaning mapping” (Steven & Plaut, 2022). These important predictions concerning statistical regularities are carefully considered in the current study’s framing and analysis such as through matching items on measures of combinatorial likelihood (Günther & Marelli, 2021, 2022; for further discussion of these measures in the context of morpheme transposition experiments, see Spencer et al., 2024). As most decompositional theories of morphological processing are underspecified regarding positional constraints (Grainger & Beyersmann, 2017), although the dual-route model incorporates distinct orthographic grain sizes (Grainger & Ziegler, 2011), the current study uses specific predictions under the word and affix model to explore the positional encoding of affixes in English (Beyersmann & Grainger, 2023). 

Specifically, the word and affix model proposes stem recognition and affix recognition as two separate mechanisms, as depicted in Fig. 1, which differ in several important aspects, including their positional constraints (Beyersmann & Grainger, 2023). While affixes are included in the orthographic lexicon with whole words, they are also positionally marked (e.g., prefixes have a place holder at the end, as in *un*_, and suffixes have a place holder at the beginning, as in _*ity*). In contrast, stem morphemes are not positionally marked and can occur in any position.

**Fig. 1:**
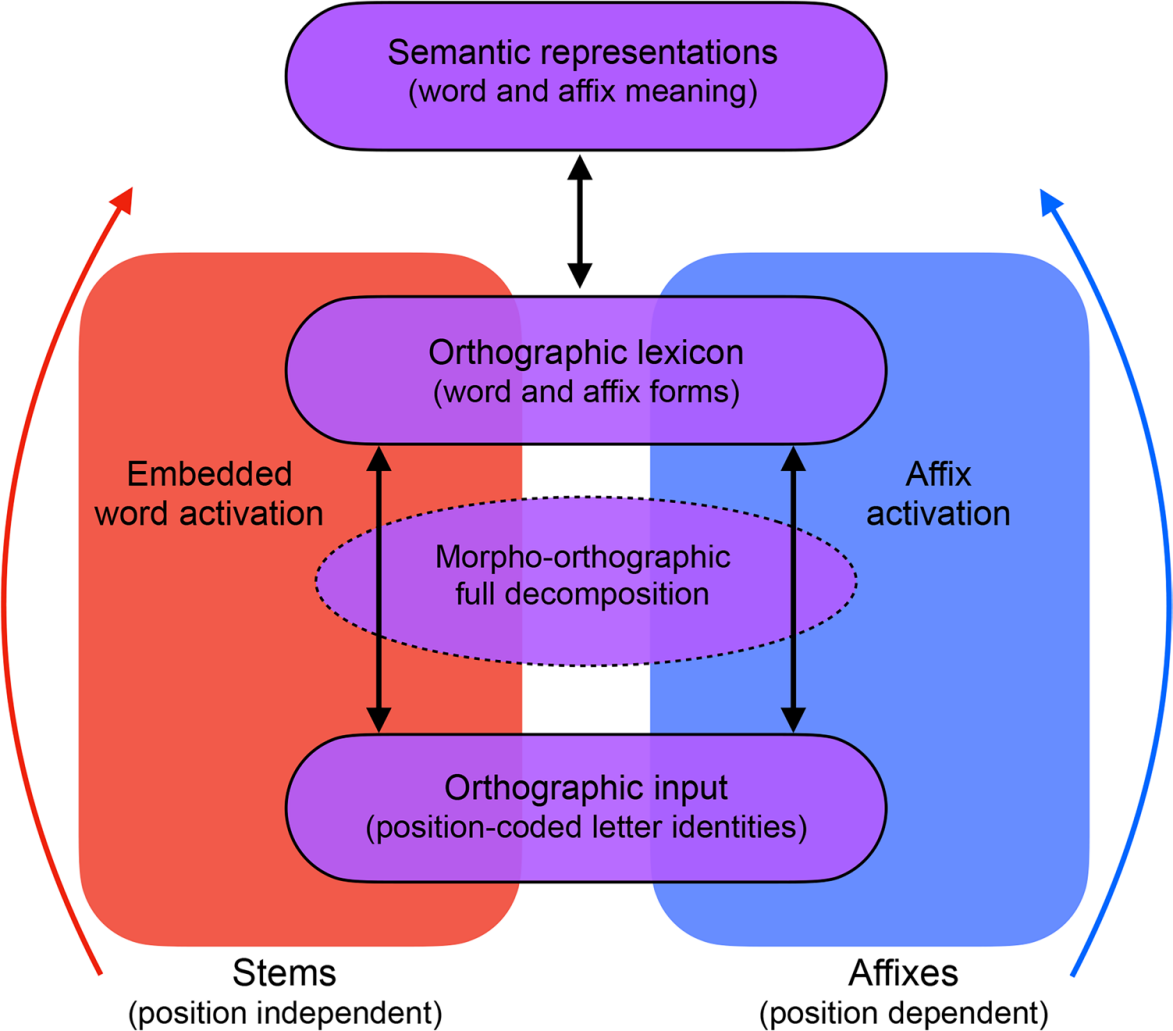
Distinct pathways for activation of stems and affixes under the word and affix model

However, the exact positional rules that the reading system applies to efficiently navigate these situations are yet to be determined. One outstanding question is how the reading system processes affixes in instances where fluent English speakers have no difficulty reading words such as *hopefulness* even though the suffix -*ful* is embedded in the middle of the letter string. The processing of complex words with mid-string embedded affixes challenges the prediction of the word and affix model that affixes must be edge-aligned. Clearly, there must be further nuance to the positional constraints imposed by the reading system on affixes, such as multiple affixes being allowed to stack on the edge of a letter string or the possibility of positional markers operating relative to stems. Thus, the specific nature of these positional encoding rules continues to be an open question with implications for models of morphological processing.

## Experiment 1

Experiment 1 explored whether suffixes are indeed identified only when embedded at the right edge of the letter string, as predicted by the word and affix model, or if suffixes are recognized in mid-embedded position (e.g., the suffix -*ful* in *spitefulness*). Items with stacked suffixes were derived from tri-morphemic words consisting of a stem followed by two suffixes (e.g., *spitefulness*), before transposing the suffixes (e.g., from *spitefulness* to *spitenessful*) and measuring interference effects relative to controls with different suffixes (e.g., *spitementdom*). The second pair of experimental conditions featured suffixes moved to initial position (e.g., *fulyouthness* from *youthfulness*), where previous studies have confirmed they will not be activated (Spencer et al., 2024), and again the controls contained different suffixes (e.g., *domyouthment*; see conditions in Table 1). If all three embedded morphemes are recognized, then items like *spitenessful* should create interference and disrupt participant responses by mapping onto the real word *spitefulness*. That is, if a morpheme transposition effect is found relative to controls (e.g., transposed stacked suffixes like *spitenessful* will be recognized more slowly and less accurately than their controls; e.g., *spitementdom*), this would indicate that suffixes can be recognized outside their typical position. Regarding the control conditions, we predicted a reduced morpheme transposition effect (e.g., a transposed separated suffix item like *fulyouthness* would be similarly difficult to reject as its control, e.g., *domyouthment*), because the suffix moved to the front will not be recognized as a morpheme. The strongest form of evidence here would be an interaction between item type (stacked suffixes/separate suffixes) and transposition (transposed/control). These predictions were preregistered along with the design and analysis plan (https://aspredicted.org/b8sa6.pdf).

**Table 1:** Experimental design

Study	Condition	Transposed	Control
Experiment 1			
	Stacked suffixes	spitenessful	spitementdom
	Initial suffix	fulyouthness	domyouthment
Experiment 2			
	Embedded suffix	bestersell	bestalsell
	Initial suffix	erwalksleep	enwalksleep

### Methods

#### Participants

Ninety-two university students (64 women; age: *M* = 21.4 years, *SD* = 7.1, min = 18, max = 54) were recruited from Macquarie University, including two extra participants to cover potential technical difficulties. A preliminary power estimate prior to commencing indicated 90 would be sufficient, conservatively assuming an effect size of 0.3 (where 0.4 is a good first estimate of the smallest effect size of interest in psychological research) with a power threshold of 80% (Brysbaert, 2019). All participants spoke English as their first language, with no reading impairments or history of neurological impairment. They were given participation credits for their time.

#### Materials

The 168 experimental items included 42 morphemically transposed stacked suffixed nonwords (e.g., *spitenessful* derived from spitefulness), as well as 42 controls (e.g., *spitementdom* where the suffixes were replaced). The second half of the nonword stimuli were comprised of 42 morphemically transposed separate suffix words (e.g., *fulyouthness* derived from youthfulness), as well as 42 controls (e.g., *domyouthment* with the suffixes replaced). In addition to these nonwords were 84 filler words with 42 items each of matched suffixed words (e.g., *objectively*, including the suffixes -*ive* and -*ly* from the nonword suffix items), and other double-suffixed words (e.g., *egotistic,* including the new suffix -*ic*). The transposed and control nonwords were matched both within and across conditions on length and word frequency (Zipf frequency retrieved from the SUBTLEX UK database; Van Heuven et al., 2014). They were also matched on position-specific bigram frequency (via the CELEX lexical database; Baayen et al., 1996), orthographic Levenshtein distance 20 (OLD20; Yarkoni et al., 2008), and letter transitional probabilities at morphemic boundaries (Marelli et al., 2013; code available at https://osf.io/yp6uq/), all of which were retrieved from the CELEX database (Baayen et al., 1996; see Table 2).

**Table 2:** Matched characteristics of Experiment 1 nonwords—Mean (standard deviation)

Variable	Stacked suffixes transposed	Stacked suffixes control	Initial suffix transposed	Initial suffix control
Length	10.76 (1.59)	10.74 (1.52)	11.28 (1.78)	11.33 (1.82)
P. bigram freq	474.76 (222.28)	482.38 (218.02)	562.26 (182.08)	542.74 (229.50)
OLD20	4.49 (0.67)	4.63 (0.64)	4.87 (0.67)	4.97 (0.78)
Original freq	2.76 (0.70)	2.76 (0.70)	2.71 (0.76)	2.71 (0.76)
Morph. bound	0.08 (0.08)	0.07 (0.08)	0.04 (0.05)	0.05 (0.07)

P. bigram freq. = mean position-specific bigram frequency; OLD20 = mean orthographic neighborhood; Original freq. = mean SUBTLEX UK Log Freq Zipf for the original word from which items were derived e.g., *spitefulness*. Morph. bound. = letter transitional probabilities at morphemic boundaries

Suffixes that did not resemble the beginning of any English words were excluded from the nonword stimuli to prevent any risk that the resulting initial suffix nonwords would be rejected quickly due to orthographically illegal beginnings (e.g., *ifyfort* from *fortify* would risk being rejected quickly because there are no English words beginning with *ify*). Suffixes that formed complete words by themselves were also excluded (e.g., -*age* and -*less*), as were inflectional suffixes (e.g., -*ing* and -*est*) and those only one letter in length (e.g., -*y*). Some suffixes that can be inflectional in some cases (e.g., the -*er* in *larger*) were still included when they are derivational in the original words that items were formed from (e.g., the derivational -*er* in *seller*). Nonword items across both experiments were also derived exclusively from semantically transparent English words (i.e., words where all constituent morphemes directly relate to the overall meaning of the word). This was important to control because distributional accounts suggest that morphological processing is essentially captured in the links between orthography and semantics, and distributional accounts including the word and affix model would expect morpheme transpositions to be facilitated by semantics (for discussion of the role of semantics in morpheme transposition effects, see Spencer et al., 2024). Due to the artificial nature of the transposed nonwords, the items were also checked for violations of English syntactic rules and matched on the number of violations across conditions that would be compared in our results (based on statistics from Plag & Baayen, 2009). Regarding stem-suffix combinations in Experiment 1, the stacked suffixes conditions have 19% (transposed) and 21% (control) violations (e.g., where the suffix *-ness* only attaches to adjectives in English but in the nonword item *assertnessive* it is attached to a verb), and the initial-suffix conditions have 24% (transposed) and 17% (control) violations.

The suffixes in the transposed nonwords (e.g., *spitenessful*/*fulyouthness*) were shuffled and then suffixes from that list were used to form the control nonwords (e.g., *spitementdom*/*domyouthment*). Items were also controlled to minimize instances of transposition changing the pronunciation of embedded constituents, and nonwords were rejected if they phonetically resembled any existing English words. Nonwords were also transposed from original words with complete stems (e.g., *globally* does not have the full stem *globe* and separates into *glob* + *al* + *ly* and would not qualify). The filler words were not held to these additional standards but were matched to the experiment items on the same factors that were matched between nonword conditions. The 168 experimental items were then split into two counterbalanced lists, and each of these halves was matched with the 84 filler items. This separation ensured that participants saw either the transposed nonword or its control, but never both, during their trials. The complete list of stimuli for Experiment 1 is included in Appendix 1.

#### Procedure

Each participant accessed the experiment online via Gorilla, where they completed the main unprimed lexical decision task and demographic survey. Participants were instructed to create a lab-like environment at home, to answer as quickly and accurately as possible, and that they must decide whether the presented letter string was a real English word or not. They first completed eight practice trials, during which they received feedback, before proceeding to the main block of trials. Each trial began with presentation of a fixation cross for 500 ms, followed by the stimulus for which participants made a lexical decision. There was a time limit of 3,000 ms on the response, where a “Yes” key was pushed for a real English word and a “No” key was pushed for a nonword. Trial presentation was randomized, and halfway through the trial block was an opportunity to take a short break. The whole experiment took approximately 12 min per participant.

#### Analyses

Analyses were performed using linear mixed-effects (LME) models (Baayen et al., 2008) as implemented in the *lme4* package (Version 1.1–27.1; Bates et al., 2015) in the statistical software R (Version 4.1.0, 2021–05–18, “Camp Pontanezen,” R Core Team, 2021). Real-word filler items were excluded from the analyses. Following Barr et al., (2013), we determined the maximal random effect structure that converged, which led us to include by-item and by-participant random intercepts. LME models were produced via the “lmer” function for reaction times and the “glmer” function for error rates. (G)lmer models both used one of the standard *lme4* optimizer’s, “bobyqa” (Bates et al., 2015). Incorrect responses were removed from the reaction time analysis, as were any responses made in less than 250 ms or after 3,000 ms. A minimal model for residual trimming of outliers was used (Baayen et al., 2008) which led to the removal of < 1% of the data. A Box-Cox transformation determined that the reaction time data did not need to be transformed. The final model contained two fixed-effect factors (item type: stacked suffix/initial suffix; transposition: control/transposed), their interactions, and random intercepts for subjects and items, and by-subject random slopes for item type and transposition. Dummy coding was used for factor item type (e.g., reference level “control”) and transposition (i.e., reference level “stacked suffix”). TrialN represents the order of trial presentation which was randomized for each participant, included to control for learning and fatigue effects. The significance of the fixed effects in our LME models was determined with Type III model comparisons using the “Anova” function in the *car* package (Version 3.0–12; Fox et al., 2019), and *p* values were obtained using the *lmerTest* package (Version 3.1–3; Kuznetsova et al., 2017). Two control items were removed for errors (*althoughtly* contains the real word *though*; *ideallyful* contains the real word *ideally*). The full LME outputs for all experiments are reported in Appendix 3. The full data and analysis files are available online (https://osf.io/yp6uq/).

## Results and discussion

**Fig. 2:**
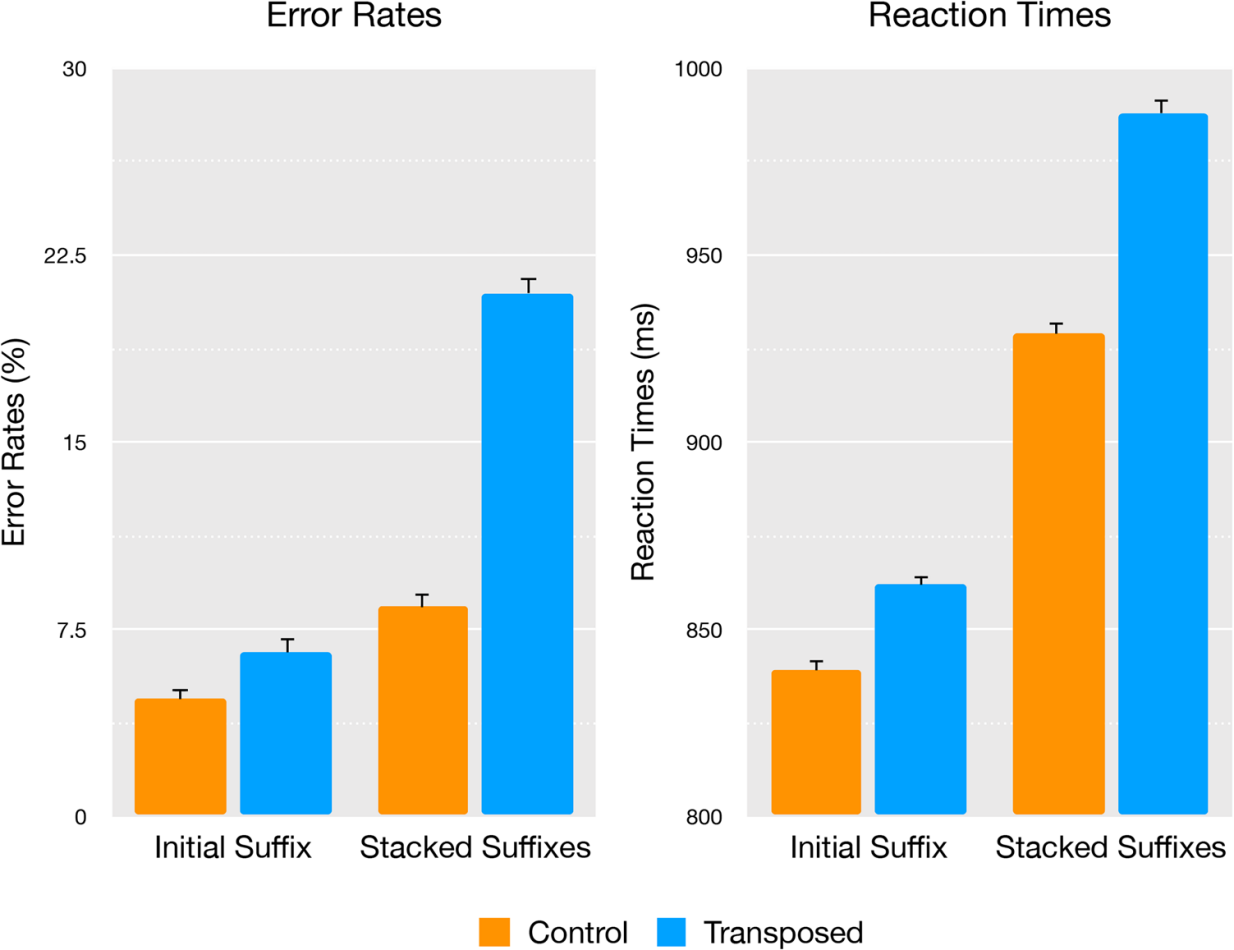
Experiment 1: Mean error rates (in %) and lexical decision times (in ms) with error bars representing standard error

The mean error rates and lexical decision reaction times for the nonword conditions are reported in Fig 2.

In the error rate analyses, the main effects of item type (stacked suffix/initial suffix), χ^2^(1) = 4.86, *p* = 0.027 and transposition (control/transposed), χ^2^(1) = 17.01, *p* ≤ 0.001, were significant, as was the interaction between these factors, χ^2^(1) = 4.01, *p* = 0.045, indicating that the effect of transposition was significant for the stacked suffix condition (*z* = 4.125, *p* < 0.001), but not for initial suffix items (*z* = 1.443, *p* = 0.149). The reaction time analyses found the same results, where the main effects of item type (stacked/initial), χ^2^(1) = 11.48, *p* ≤ 0.001 and transposition (control/transposed), χ^2^(1) = 8.13, *p* ≤ 0.004, were significant, as was the interaction between these factors, χ^2^(1) = 4.59, *p* = 0.032, showing that the effect of transposition was significant for stacked suffix items (*t* = 2.85, *p* = 0.006), but not for initial suffix items (*t* = 0.79, *p* = 0.430).

As expected, transposed stacked suffix nonwords (e.g., *spitenessful*) took longer to reject (*M* = 988.36, *SD* = 475.19) and yielded much higher error rates (*M* = 20.96%, *SD* = 40.71%) than their control counterparts’ (e.g., *spitementdom*) reaction times (*M* = 929.34, *SD* = 408.84) and error rates (*M* = 8.36%, *SD* = 27.69%). Conversely, transposed initial suffix nonwords (e.g., *fulyouthness*) took a similar amount of time to reject (*M* = 862.08, *SD* = 375.20) compared with their control counterparts (e.g., *domyouthment*) (*M* = 839.19, *SD* = 366.55) and showed no significant difference in error rates between transposed (*M* = 6.65%, *SD* = 24.92%) and control (*M* = 4.70%, *SD* = 21.16%) conditions.

The results of Experiment 1 show morpheme transposition effects in tri-morphemic words with mid-embedded suffixes. There are two different explanations for this finding. One possibility is that mid-embedded suffixes are recognized because they follow a stem. That is, as long as suffixes do not occur in string-initial position (like -*ful* in *fulyouthness*) and either directly follow a stem (like -*ness* in *spitenessful*) or occur in string-final position (like -*ful* in *spitenessful*), the embedded morphemes are successfully identified and mapped onto the corresponding lexical whole-word representation (i.e., *spitefulness*). Alternatively, mid-embedded suffixes may be recognized without being attached to a stem, as long as they are part of a chain of suffixes stacked at the end of a letter string (i.e., they follow all stems in the word, as in *humanitarianism*). This latter option would predict that the visual recognition of complex words is constrained in the sense that suffixes must be either edge-aligned (e.g., -*ful* in *spitenessful*) or directly precede another suffix (e.g., -*ness* in *spitenessful*). Whether either of these positional constraints apply to suffixes was addressed in Experiment 2.

## Experiment 2

Experiment 2 explored whether the identification of mid-embedded suffixes depends only on them following a stem, or also requires this suffix morpheme being “stacked” as part of a chain of suffixes on the end of a letter string, by testing items with a mid-embedded suffix that follows a stem but does not form part of a chain of suffixes. Embedded suffix items were derived from tri-morphemic words consisting of two stems followed by a suffix (e.g., *bestseller*), before transposing the last two morphemes and measuring interference effects relative to controls (e.g., *bestersell*/*bestalsell*). The second pair of experimental conditions featured suffixes moved to initial position (e.g., *erwalksleep*/*enwalksleep*), where previous studies have confirmed it will not be activated (Spencer et al., 2024), and again the controls contained different suffixes (e.g., *enwalksleep*; see conditions in Table 1). Stem morphemes in the initial suffix conditions were transposed (e.g., *walksleep* in *erwalksleep*) to better resemble the embedded suffix nonwords (e.g., *bestersell*) that do not have their two stem morphemes connected either.

If a morpheme transposition effect is found for nonwords including mid-embedded suffixes (e.g., transposed embedded suffixes like *bestersell* will be recognized more slowly and less accurately than their controls e.g., *bestalsell*), this would indicate that *bestersell* was able to map onto the real word *bestseller* because suffixes can be recognized when neither edge-aligned nor forming part of a chain of string-final suffixes. Regarding the initial suffix control condition, we predicted a reduced morpheme transposition effect (e.g., a transposed initial suffix item like *erwalksleep* would be similarly difficult to reject as its control, e.g., *enwalksleep*), because the suffix moved to the front will not be recognized as a morpheme. The strongest form of evidence here would be an interaction between item type (embedded suffix/initial suffix) and transposition (transposed/control). These predictions were preregistered along with the design and analysis plan (https://aspredicted.org/et5pm.pdf).

### Methods

#### Participants

Ninety university students (62 women; age: *M* = 24.3 years, *SD* = 10.5, min = 17, max = 65) were recruited from Macquarie University. A preliminary power estimate prior to commencing indicated 90 would be sufficient, conservatively assuming an effect size of 0.3 (where 0.4 is a good first estimate of the smallest effect size of interest in psychological research) with a power threshold of 80% (Brysbaert, 2019). All participants spoke English as their first language, with no learning impairments or history of neurological impairment. They were given participation credits for their time.

#### Materials

The 160 experimental items included 40 morphemically transposed embedded suffix nonwords (e.g., *bestersell* derived from *bestseller*), as well as 40 controls (e.g., *bestalsell* where the embedded suffix is replaced). The second half of the nonword stimuli were comprised of 40 morphemically transposed initial suffix nonwords (e.g., *erwalksleep* derived from *sleepwalker*), as well as 40 controls (e.g., *enwalksleep* where the initial suffix is replaced). In addition to these nonwords were 80 filler words with 40 items each of matched suffix words (e.g., *dogwalker*) and other suffixed words (e.g., *airhostess*). Items were matched on the same measures and carefully included/rejected according to the same criteria as in Experiment 1 (see Table 3), and were matched for stem-suffix syntactic violations where the embedded-suffix conditions had 15% (transposed) and 22% (control) violations (Plag & Baayen, 2009). As in Experiment 1, the 160 experimental items were then split into two counterbalanced lists and each of these halves was matched with the 80 filler items. The complete list of stimuli for Experiment 2 is included in Appendix 2.

**Table 3:** Matched characteristics of Experiment 2 Nonwords—Mean (standard deviation)

Variable	Embedded suffix transposed	Embedded suffix control	Initial suffix transposed	Initial suffix control
Length	10.53 (1.01)	10.60 (0.87)	10.93 (0.97)	10.95 (0.88)
P. bigram freq	491.98 (233.01)	420.60 (208.31)	258.60 (237.44)	257.03 (207.87)
OLD20	4.64 (0.71)	4.77 (0.49)	5.50 (0.75)	5.47 (0.67)
Original freq	2.46 (0.78)	2.46 (0.78)	2.35 (0.60)	2.35 (0.60)
Morph. bound	0.17 (0.09)	0.14 (0.10)	0.04 (0.05)	0.05 (0.07)

P. bigram freq. = mean position-specific bigram frequency; OLD20 = mean orthographic neighborhood; Original freq. = mean SUBTLEX UK Log Freq Zipf for the original word from which items were derived e.g., *spitefulness*. Morph. bound. = letter transitional probabilities at morphemic boundaries

#### Procedure

The procedure was identical to that of Experiment 1.

#### Analyses

Analyses followed the general scheme of those in Experiment 1. The final model contained two fixed-effect factors (item type: embedded suffix/initial suffix; transposition: control/transposed), their interactions, and random intercepts for subjects and items, and by-subject random slopes for item type and transposition. Dummy coding was used for the two fixed effects, including factor item type (e.g., reference level “control”) and for factor transposition (i.e., reference level “embedded suffix”). Two control items were removed for resemblance to real English words (*steeledwork* contains the real word *steeled*; *haireddress* contains the real word *haired*). The full LME outputs for all experiments are reported in Appendix 3. The only factor that was not matched across all four conditions in Experiment 2 was positional bigram frequency, although it was still matched within both embedded suffix and initial suffix transposed/control condition pairs to ensure it does not account for any morpheme transposition effect found between these experimental and control conditions. This is one of the reasons that direct comparisons in our analyses were not made between the transposed embedded suffix and transposed initial suffix conditions, and instead we compared the differences between the transposed and control condition pairs (that are matched on positional bigram frequency). Exploratory analyses (that were not preregistered) were run after data collection that included positional bigram frequency as a covariate and confirmed it was not responsible for the effects found (also included in Appendix 3). The full data and analysis files are available online (https://osf.io/yp6uq/).

## Results and discussion

**Fig. 3:**
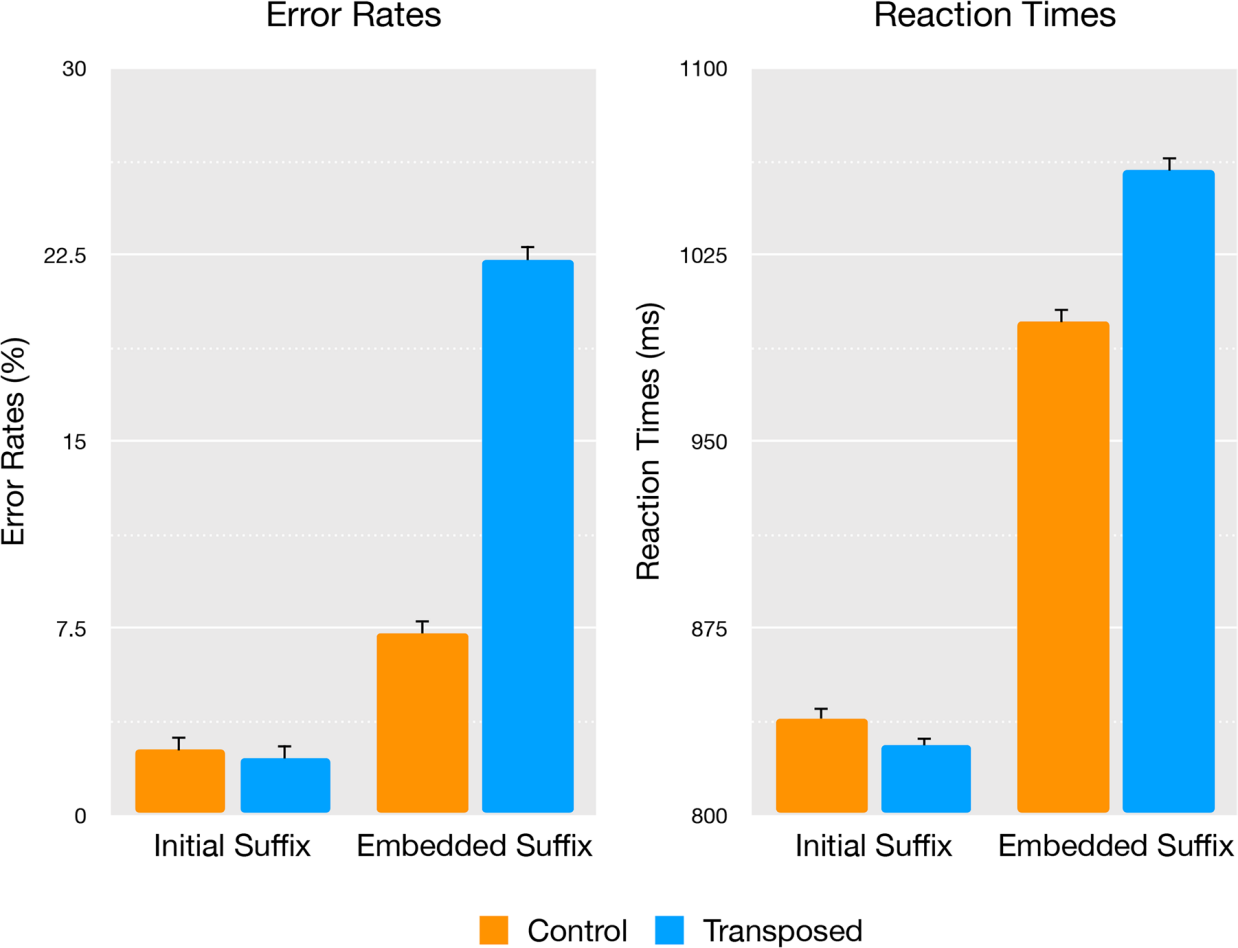
Experiment 2: Mean error rates (in %) and lexical decision times (in ms) with error bars representing standard error

The mean error rates and lexical decision reaction times for the nonword conditions are reported in Fig 3.

In the error rate analyses, the main effects of item type (embedded suffix/initial suffix), χ^2^(1) = 23.12, *p* ≤ 0.001 and transposition (control/transposed), χ^2^(1) = 57.25, *p* ≤ 0.001, were significant, as was the interaction between these factors, χ^2^(1) = 23.12, *p* ≤ 0.001, indicating that the effect of transposition was significant for the embedded suffix condition (*z* = 7.57, *p* < 0.001), but not for initial suffix items (*z* = − 0.50, *p* = 0.620). The reaction time analyses found the same results, where the main effects of item type (embedded/initial), χ^2^(1) = 56.96, *p* ≤ 0.001 and transposition (control/transposed), χ^2^(1) = 20.05, *p* ≤ 0.001 were significant, as was the interaction between these factors, χ^2^(1) = 21.00, *p* ≤ 0.001, showing that the effect of transposition was significant for embedded suffix items (*t* = 4.48, *p* < 0.001), but not for initial suffix items (*t* = − 1.27, *p* = 0.209). Additionally, the effect of positional bigram frequency was not significant for error rates χ^2^(1) = 1.02, *p* = 0.312, or reaction times χ^2^(1) = 0.54, *p* = 0.461.

As expected, transposed embedded suffix nonwords (e.g., *bestersell*) took longer to reject (*M* = 1059.02, *SD* = 452.32) and yielded much higher error rates (*M* = 22.3%, *SD* = 41.64%) than their control counterparts (e.g., *bestalsell*) reaction time (*M* = 998.34, *SD* = 412.06) and error rates (*M* = 7.3%, *SD* = 26.0%). Conversely, transposed initial suffix nonwords (e.g., *erwalksleep*) took a similar amount of time to reject (*M* = 828.49, *SD* = 357.12) compared to their control counterparts (e.g., *enwalksleep*) (*M* = 839.31, *SD* = 368.63) and showed no difference in error rates between transposed (*M* = 2.3%, *SD* = 15.0%) and control (*M* = 2.6%, *SD* = 15.8%) conditions.

The results of Experiment 2 show that the morpheme transposition effects extend to tri-morphemic words with mid-embedded suffixes that are neither edge-aligned nor stacked in a chain of suffixes at the end of the letter string. This indicates greater flexibility in recognition of affix morphemes than predicted by the word and affix model, so potentially the only positional constraint is for suffixes to not occur at the onset of words but otherwise be freely recognized.

## General discussion

The present lexical decision study investigated the positional constraints of suffixes during visual word recognition within two experiments. Experiment 1 examined morpheme transpositions within tri-morphemic words consisting of a stem and two suffixes (e.g., *spitefulness*), comparing four conditions: a transposed suffix condition where the positions of the two word-final suffixes were transposed (e.g., *spitenessful*), a transposed suffix control condition (e.g., *spitementdom*), an initial suffix condition where the mid-embedded suffix was moved into initial position (e.g., *fulyouthness*), and an initial suffix control condition (e.g., *domyouthment*). Experiment 2 examined morpheme transpositions within tri-morphemic words consisting of two stems and a suffix (e.g., *bestseller*), comparing four conditions: an embedded suffix condition, where the suffix was moved into mid-embedded position (e.g., *bestersell*); an embedded suffix control condition (e.g., *bestalsell*); an initial suffix condition, where the suffix was moved into initial position (e.g., *erwalksleep*); and an initial suffix control condition (e.g., *enwalksleep).*

**Fig. 4:**
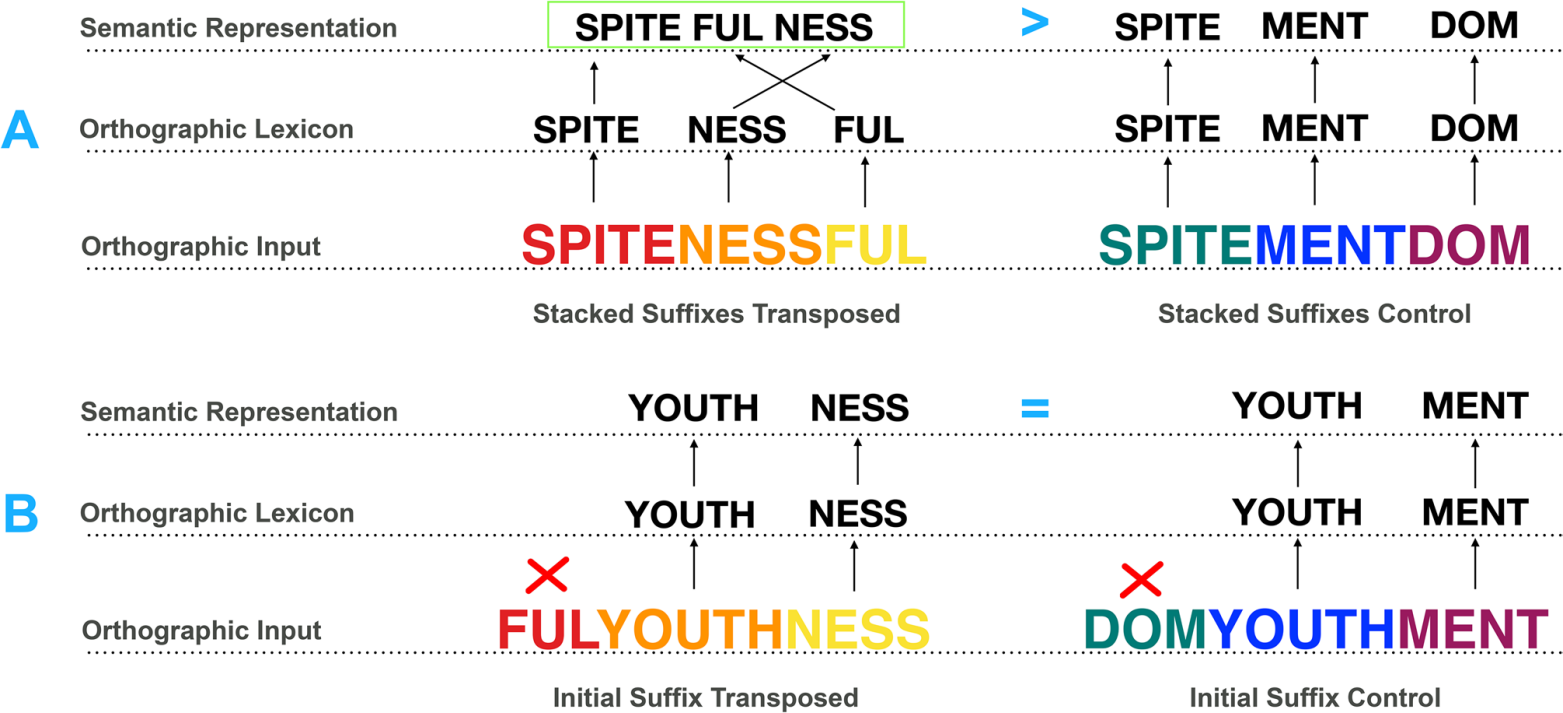
Experiment 1—Morpheme transposition effects by condition

Experiment 1 revealed mid-embedded suffixes are meaningfully recognized, as evidenced by items such as *spitenessful* causing interference relative to their controls (as in Fig. 4). The observed morpheme transposition effect confirmed that readers were able to identify all three morphemes, suggesting that recognition of mid-embedded suffixes as morphemes is possible despite them not being edge-aligned. This clearly challenges the assumption that suffixes must be bound to the right edge of the letter string (Beyersmann & Grainger, 2023).

**Fig. 5:**
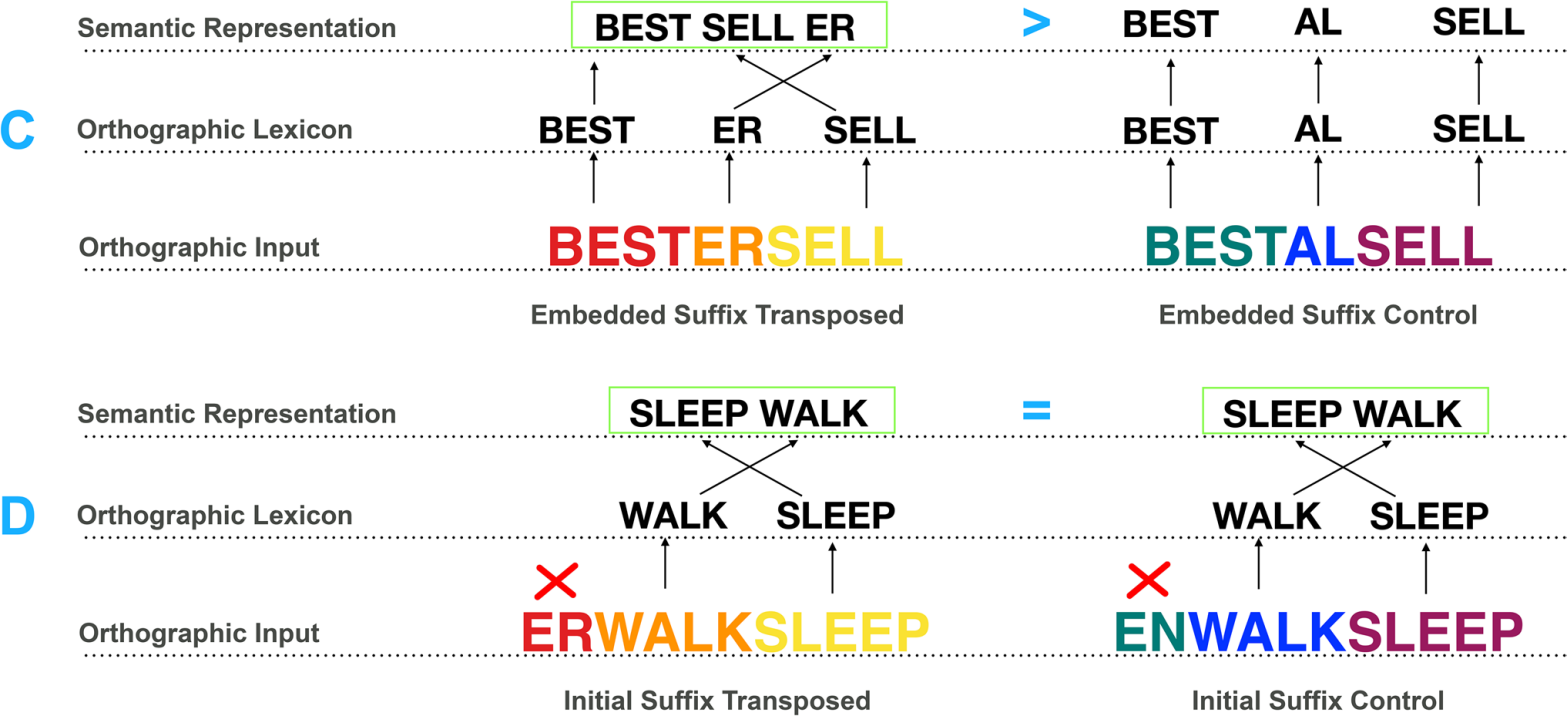
Experiment 2—Morpheme transposition effects by condition

The results of Experiment 2 further clarify the positional constraints of affix processing in reading, based on findings that items such as *bestersell* can also produce interference effects relative to controls (Fig. 5). While items like *spitenessful* in Experiment 1 were formed following one of the key principles of English morphology using two suffixes in suffix-position (as in *care-ful-ly* and *end-less-ly*), the items included in Experiment 2 had an atypical linguistic structure with mid-embedded suffixes that preceded another stem. While compounding in other Indo-European languages such as German commonly includes infixes (such as the linking suffix *s* in *Liebe-s-lied* [love song] or *Arbeit-s-zimmer* [home office]), the use of infixes in English is highly uncommon (with a few rare exceptions such as *passerby*). The results of Experiment 2 suggest that even when occurring in a linguistically unlikely middle position (*best* + *er* + *sell*), separated from a meaningful stem (i.e., *bester* is not a word), and without being stacked in a chain of affixes at the end of the letter string, suffixes can still be recognized as morphemes.

The present findings are difficult to account for under decompositional and distributional theories of morphological processing that do not incorporate any explicit positional rules for morpheme recognition. Crucially, the items in the current study were matched on measures of combinatorial likelihood and word-likeness (e.g., position-specific bigram frequency and letter transitional probabilities at morphemic boundaries; Duñabeitia et al., 2014) to ensure these factors were not driving any effects that emerged. One of the few theoretical frameworks that makes specific predictions regarding the positional encoding of morphemes is the word and affix model (Beyersmann & Grainger, 2023), which we aimed to explore in this study. The word and affix model uses three distinct mechanisms; nonmorphological embedded word activation, morphological affix activation, and full morpho-orthographic decomposition (Beyersmann & Grainger, 2023). The parallel embedded word and affix activation mechanisms associate orthography with meaning (see Fig. 1), while incorporating the distinct status of stems and affixes in the reading system. This makes the word and affix model one of very few to base predicted activation patterns of stems and affixes on consideration of their different positional constraints and orthographic grain sizes (Grainger & Beyersmann, 2017, 2021). More specifically the model expects that the reading system avoids activating affixes in the wrong position (e.g., -*er* in *error*) because affix representations are tagged with positional markers in the orthographic lexicon (e.g., *_ity, _er*). Crucially, this general expectation for affixes to be edge-aligned was challenged by the current data, and thus calls for a modification of the principle of edge-alignment within the word and affix model, and highlights the need for the further specification of morpheme-position constraints in visual word recognition theories more generally.

One possible explanation for the current findings is that the relative importance of edge-alignment differs depending on whether readers are processing an exhaustively decomposable letter string like *farmer,* or a nonexhaustively decomposable letter string like *cashew.* Whenever letter strings are exhaustively decomposable into morphemic subunits, such as the items in the current study (e.g., *best* + *er* + *sell*), the principle of morpho-orthographic full decomposition will operate on the active subunits within the orthographic lexicon (Beyersmann & Grainger, 2023) with tolerance for orthographically underspecified embedded words (e.g., *ador* in *adorable*). Our findings suggest that the assumptions of the word and affix model need to be tweaked such that edge-aligned embedded word activation is only successful if the embedded word is either (i) accompanied either by one or multiple suffixes to its right edge (as in *spitenessful*), or (ii) a suffix and another embedded word to its right edge (as in *bestseller*). If both these assumptions are met, then a word is activated that matches the length of the input (e.g., *spitenessful* activates *spitefulness*, or *bestersell* activates *bestseller*).

In conclusion, the present findings demonstrate that positional encoding of suffixes in English is more flexible than previously assumed. Although string-initial suffix recognition clearly fails, mid-embedded suffixes appear to be easily identified, thereby challenging claims that suffixes must be edge-aligned or cannot precede stems. These findings align with the notion that position-independent stems and (relatively) position-dependent affixes occupy distinct roles in complex visual word recognition (Spencer et al., 2024), while further clarifying the nature of positional constraints on English morphemes and calling for more nuanced theoretical models of morphological processing.

## Data Availability

The datasets and materials for all experiments are available in the OSF repository at (https://osf.io/yp6uq/) and both Experiment 1 (https://aspredicted.org/b8sa6.pdf) and Experiment 2 (https://aspredicted.org/et5pm.pdf) were preregistered.

## Appendix 1

Tables 4, 5, 6 and 7.

**Table 4:** Complete list of target stimuli used in Experiment 1 (excluding real word filler items)

Transposed Stacked Suffixes	Control Stacked Suffixes	Transposed Initial Suffix	Control Initial Suffix
respectlyive	respectistal	iveprogressly	ousprogressity
masslyive	massicish	iveeffectly	aleffectness
instinctlyive	instinctistal	ivecollectly	fulcollectity
actlyive	actnessish	iveimpressly	fulimpressist
westerern	westlyful	ernnorther	ousnorthly
freaklyish	freaknessern	ishfoolly	ivefoolness
selflyish	selfnessous	ishchildly	fulchildity
devillyish	devilical	ishfeverly	ualfeverness
spiritlyual	spiritnessal	ualintellectly	fulintellectist
nationistal	nationlyic	altraditionist	isttraditionly
methodalic	methodlyful	icmythal	fulmythly
articist	artalish	istmoralic	ivemoralness
materialicist	materialerous	istritualic	iveritualic
idealicist	ideallyful*	istrealic	ousrealness
assertnessive	assertlyful	iveattractness	alattractly
expressnessive	expresslyal	altraditionly	fultraditionly
personityal	personlyful	ivedistinctness	aldistinctly
musicityal	musiclyish	aloriginity	icoriginly
nationityal	nationlyful	alfunctionity	fulfunctionly
protectistion	protectlyness	ionperfectist	fulperfectly
expressistion	expresslyual	ionimpressist	alimpressly
eventityual	eventlyish	ualspiritity	ishspiritly
zeallyous	zealnessist	ousscandally	ionscandally
perillyous	perilistful	ousjoyly	ernjoyness
childnessish	childlyful	ishboyness	fulboyly
foolnessish	foolistful	alfunctionly	fulfunctionly
criticlyal	criticnessful	ishdevilness	fuldevilly
ethiclyal	ethicerness	alclassicly	icclassicness
conventionlyal	conventionical	allogicly	istlogicity
cliniclyal	clinicnessist	alaccidently	istaccidentity
digitlyal	digiterist	altacticly	fultacticness
regionlyal	regionityive	alseasonly	fulseasoner
gracelyful	gracenessal	alfantasticly	ishfantasticly
carelyful	carenessern	fulfaithly	ivefaithal
forcelyful	forcelyal	fulcheerly	ishcheeric
respectlyful	respectlyion	fulwoely	alwoely
usenessful	uselyive	fulpowerly	ishpoweric
forgetnessful	forgetlyion	fultruthness	altruthly
youthnessful	youthlyal	fulplayness	ualplayly
peacenessful	peacelyal	fulhopeness	ivehopely
joynessful	joylyive	fulthoughtness	althoughtly*
mindnessful	mindlyal	fulwasteness	ivewastely

Items with an asterisk (*) were removed for reasons described in the corresponding results

## Appendix 2

**Table 5:** Complete list of target stimuli used in Experiment 2 (excluding real word filler items)

Transposed Embedded Suffix	Control Embedded Suffix	Transposed Initial Suffix	Control Initial Suffix
bestersell	bestivesell	erworkcase	lyworkcase
absentedmind	absentlymind	erleadcheer	edleadcheer
antereat	antnesseat	ermailblack	enmailblack
bloodersuck	bloodedsuck	erlaybrick	edlaybrick
bodyerbuild	bodyedbuild	erbindbook	ernbindbook
bullerfight	bullenfight	ercastbroad	encastbroad
clifferhang	cliffenhang	ersellbook	edsellbook
dayerdream	dayaldream	erfindview	nessfindview
demoicgraph	demoedgraph	erneastsouth	ereastsouth
disherwash	dishenwash	ercrackfire	edcrackfire
doorerkeep	doorenkeep	erthrowflame	edthrowflame
footerball	footedball	ercatchfly	ismcatchfly
goalerkeep	goaledkeep	edfootsure	ivefootsure
hairerdress	haireddress*	lymangentle	ermangentle
handenmaid	handermaid	erblowglass	ernblowglass
painerkill	painalkill	erownhome	ishownhome
metalerwork	metaledwork	erownland	ernownland
nutercrack	nutalcrack	erspeakloud	edspeakloud
otherlyworld	othererworld	ernwestnorth	erwestnorth
patherfind	pathedfind	ernwestsouth	erwestsouth
standishoff	standedoff	erneastnorth	ereastnorth
tablefulspoon	tableerspoon	ergraphphoto	engraphphoto
wooderwork	woodalwork	ismrealphoto	errealphoto
homenesssick	homefulsick	erfightprize	edfightprize
shoperlift	shopishlift	nessthingno	erthingno
teafulspoon	tealyspoon	ishboytom	ernboytom
steelerwork	steeledwork*	iveactradio	eractradio
deererstalk	deerenstalk	erbreaktie	nessbreaktie
storyertell	storyedtell	enfallcrest	erfallcrest
taxerpay	taxnesspay	edheartwhole	erheartwhole
streeterwalk	woodalpeck	nesssicksea	ersicksea
weekerend	weekeryend	ergraphradio	engraphradio
coldedheart	coldenheart	erholdshare	enholdshare
wooderpeck	woodalpeck	ershootsharp	edshootsharp
fortlynight	forternight	erkeepstore	edkeepstore
wrongedhead	wrongerhead	edsightshort	ersightshort
breakedfast	breakalfast	erboardskate	enboardskate
bigedhead	bigerhead	erwalksleep	enwalksleep
tomeryfool	tomfulfool	erholdstake	edholdstake
withaldraw	withicdraw	erdowrong	erndowrong

Items with an asterisk (*) were removed for reasons described in the corresponding results. ***breakedfast* was erroneously included despite being derived from an original word containing an inflectional suffix, but was ultimately included due to the small number of English words possible to use for the paradigm needed in Experiment 2

## Appendix 3

**Table 6:** Linear mixed-effects model output anova for Experiment 1

Variable	Chisq	*df*	*p* value	Sign
Error rates				
(Intercept) Item type Transposition Scale(TrialN) Item type: Transposition	183.5483 4.8619 17.0128 2.2034 4.0149	1 1 1 1 1	< 0.001 0.027 < 0.001 0.137 0.045	*** * *** *
Reaction Times				
(Intercept) Item type Transposition Scale(TrialN) Item type: Transposition	754.8146 11.4822 8.1346 309.9736 4.5864	1 1 1 1 1	< 0.001 < 0.001 < 0.004 < 0.001 0.032	*** *** ** *** *

Significance: * = < 0.05 ** =  < 0.01 *** =  < 0.001. Item type = stacked or initial

**Table 7:** Linear Mixed-Effects Model Output Anova for Experiment 2

Variable	Chisq	*df*	*p* value	Sign
Error rates				
(Intercept) Item type Transposition Scale(TrialN) Item type: Transposition Positional Bigram Frequency	249.5659 23.1166 57.2483 3.1425 23.1203 1.0226	1 1 1 1 1 1	< 0.001 < 0.001 < 0.001 0.076 < 0.001 0.312	*** *** *** * ***
Reaction Times				
(Intercept) Item type Transposition Scale(TrialN) Item type: Transposition Positional Bigram Frequency	1028.026 56.958 20.045 342.427 21.001 0.5446	1 1 1 1 1 1	< 0.001 < 0.001 < 0.001 < 0.001 < 0.001 0.461	*** *** *** *** ***

Significance: * = < 0.05 ** =  < 0.01 *** =  < 0.001. Item type = embedded or initial

## Code availability

Scripts for data analysis are also available at (https://osf.io/yp6uq/).
